# Layered Double Hydroxides as Systems for Capturing Small-Molecule Air Pollutants: A Density Functional Theory Study

**DOI:** 10.3390/molecules29214996

**Published:** 2024-10-22

**Authors:** Elaheh Mohebbi, Cristina Minnelli, Eleonora Pavoni, Laura Sisti, Emiliano Laudadio, Pierluigi Stipa

**Affiliations:** 1 Department of Science and Engineering of Matter, Environment and Urban Planning, Polytechnic University of Marche, 60121 Ancona, AN, Italy; e.mohebbi@staff.univpm.it (E.M.); e.pavoni@staff.univpm.it (E.P.); p.stipa@staff.univpm.it (P.S.); 2 Department of Life and Environmental Sciences, Polytechnic University of Marche, 60121 Ancona, AN, Italy; c.minnelli@staff.univpm.it; 3 Department of Civil, Chemical, Environmental and Materials Engineering, University of Bologna, 40126 Bologna, BO, Italy; laura.sisti@unibo.it

**Keywords:** DFT, LDH, air pollutants, density of states, charge transfer

## Abstract

Air pollutants are usually formed by easily spreading small molecules, representing a severe problem for human health, especially in urban centers. Despite the efforts to stem their diffusion, many diseases are still associated with exposure to these molecules. The present study focuses on modeling and designing two-dimensional systems called Layered Double Hydroxides (LDHs), which can potentially trap these molecules. For this purpose, a Density Functional Theory (DFT) approach has been used to study the role of the elemental composition of LDHs, the type of counterion, and the ability of these systems to intercalate NO_2_ and SO_2_ between the LDH layers. The results demonstrated how the counterion determines the different possible spacing between the layers, modulating the internalization capacity of pollutants and determining the stability degree of the system for a long-lasting effect. The variations in structural properties, the density of states (DOS), and the description of the charge transfer have been reported, thus allowing the investigation of aspects that are difficult to observe from an experimental point of view and, at the same time, providing essential details for the effective development of systems that can counteract the spread of air pollutants.

## 1. Introduction

Air pollutants are substances that alter the natural air chemical composition, with significant consequences for both human health and the environment. Many different governments and research centers increasingly recognize the relevance of outdoor and indoor air pollution as the main environmental risk factor for global health, as it is responsible for an estimated 3 million deaths annually [1]. Over 92% of the world’s population still live in areas with pollutant concentrations exceeding the levels recommended by the WHO Air Quality Guidelines [2]. Furthermore, the IARC (International Agency for Research on Cancer) has classified outdoor air pollution as carcinogenic to humans (Group 1) [3].

In most urban areas, the main contributor to air pollution is vehicular traffic, and industrial production can drastically increase emissions. However, while the impact of vehicles has sharply fallen over the years, biomass-fueled plants now contribute significantly to air pollution, and their emissions have increased by 113% since 1990 [4,5]. This growth is largely uncontrolled and favored by economic incentives aimed at promoting renewable energy systems. Demographic and urban development has generally led to the emergence of large industrial areas near urban centers, whose emissions have increased overall air pollution levels. However, technological developments (e.g., Best Available Techniques—BATs), combined with more strict legislation, have enabled the industrial sector to significantly reduce its impact on air quality in recent years. In fact, the BATs applied to industrial plants, the use of “cleaner” fuels (with a low sulfur content), reductions in vehicular emissions, air quality monitoring networks, and recovery plans aligned with local legislation are all crucial for counteracting the release of pollutants [6,7].

Despite the enormous efforts made to limit exposure, the ease with which small molecules form air pollutants represents a crucial problem and a major challenge for both research and public health. Each pollutant exhibits distinct chemical and physical properties, areas of probable accumulation, sources of emissions, effects on health, and critical periods throughout the year [8,9]. Therefore, research can be focused on specific classes of compounds to develop systems that are able to capture them. Among the most hazardous classes of pollutants are nitrogen oxides [10], i.e., nitrogen monoxide (NO) and nitrogen dioxide (NO_2_). NO is an odorless and colorless gas that represents the main component of nitrogen oxide emissions in the air and gradually oxidizes into NO_2_, a red-brown gas with a pungent, suffocating odor at high concentrations. The danger of inhaling nitrogen oxides, particularly NO_2_, is linked to their role in the formation of photochemical smog [11,12]. Under stable meteorological conditions and high amounts of solar radiation (spring and summer), ultraviolet radiation can cause NO_2_ dissociation and the formation of ozone, which, in turn, can recombine with NO, re-establishing an equilibrium. The health effects of NO_2_ are approximately four times more severe than NO. However, the biochemical mechanisms by which NO_2_ induces its toxic effects are unclear; it is known that it causes severe damage to cell membranes through the oxidation of proteins and lipids. The most critical effects include inflammation of the mucous membranes, decreased lung function, pulmonary edema, pulmonary alterations at cellular and tissue levels, and increased susceptibility to bacterial and viral pulmonary infections.

Another significant class of air pollutants is sulfur oxides (SO_x_), which essentially consist of sulfur dioxide (SO_2_) and, to a lesser extent, sulfur trioxide (SO_3_). SO_2_ is a gas with a characteristic pungent odor that easily interacts with nucleic acids, proteins, lipids, and various other biological components [13,14]. These pollutants typically accumulate in urban and industrial areas, promoted by high population densities, especially in meteorological conditions with limited air mass exchange [15,16]. Thanks to the widespread shift to natural gas for domestic heating, the contribution of SO_x_ to air pollution has significantly decreased in recent years [17,18]. Due to its high solubility in water, SO_2_ is easily absorbed by the mucous membranes of the nose and the upper respiratory tract [19]. Health effects associated with exposure to high levels of SO_2_ include increased airway resistance due to the swelling of mucous membranes, an increase in mucous secretions, bronchitis, tracheitis, and bronchial spasms.

**Figure 1:**
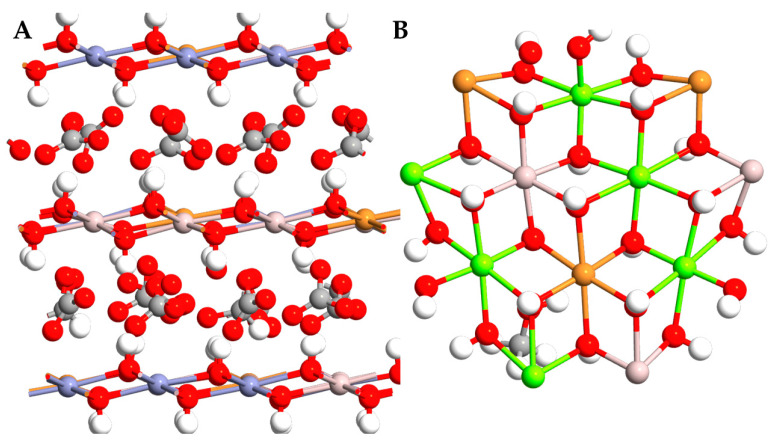
Front view (**A**) and top view (**B**) of an LDH system. Al, Cu, Zn, Mg, O, C, and H atoms are colored in pink, orange, purple, green, red, grey, and white, respectively.

Considering these critical aspects, this study aims to model and design promising advanced functional inorganic systems that can detect and trap NO_2_ and SO_2_, thereby decreasing their concentrations in the environment. The current research focuses on active bidimensional systems based on earth-abundant metals; within this framework, copper (Cu)-based Layered Double Hydroxides (LDHs) represent the most promising material. Cu is an abundant metal that does not pose a risk of carbon monoxide poisoning, making it an ideal choice for this application [20]. LDHs are a class of inorganic materials with hydrotalcite-like lamellar structures composed of divalent and trivalent metal hydroxides. Specifically, the isomorphous substitution of some divalent cations by trivalent cations leads to excess positive charges, counterbalanced by some interlayer anions and water, resulting in a neutral structure (Figure 1) [21]. The general formula of such so-called hydrotalcite-like compounds is [M^II^*_1-x_*M^III^*_x_*(OH)_2_]*^x^*^+^(A*^n^*^−^)*^x/n^ m*H_2_O, wherein M^II^, M^III^, and A^n−^ denote divalent cations, trivalent cations, and the interlayer anion, respectively. A wide variety of organic anions can be interspersed between the lamellae to modify the hydroxyl platelets and adapt them to specific end uses. This class of systems is inexpensive, easy to synthesize in high quantities (even more than gram-scale), and highly customizable as different metals and metal ratios can be considered, along with various interleaved molecules and/or anions [22]. A key advantage of LDHs is their versatility, as they can act either in solutions, for water decontamination [23], or as solid systems in contact with the atmosphere, making them prominent candidates for air purification. Due to their potential for interposing different types of molecules and macromolecules [24] LDHs are optimal candidates for capturing and trapping various pollutants.

This functional property is strongly influenced by the host anion that resides between the layers and cations in the basal layer, as well as the interlayer spacing and the stability of the architecture. The ability to tune the LDH structure is an essential issue in the research of LDH materials. For this reason, an atomistic simulation approach based on Density Functional Theory (DFT) was used for a detailed investigation of the lattice parameters of the two different copper-based LDHs, Cu-Zn-Al and Cu-Mg-Al LDH systems, in the presence of OH^−^ and CO_3_^2−^ ions as counter components. Among the various theoretical approaches, DFT is a key tool in the rapid expansion of many research fields, including the investigation of the chemical reactivity of complex systems and the simulation of intercalation of pollutants, because it facilitates the understanding of complex chemical processes at the molecular and atomic levels. This means that the use of DFT calculations in air pollution control is growing [23,24,25]. The intercalation of the NO_2_ and SO_2_ pollutants has been measured by considering the ability of these molecules to replace water and counterions possibly trapped between the layers. Therefore, although from an experimental point of view, small quantities of water can be retained between the layers, in this work the scenario in which the LDH systems are saturated with air pollutants has been considered, thus ignoring each water molecule and counter-region after the intercalation of NO_2_ and SO_2_. In this perspective, the attractive and repulsive phenomena between molecules and LDH have been investigated. The ability of LDH to trap pollutants has also been analyzed by considering the variation in structural properties, bandgap and density of states (DOS), and charge transfer distribution. The results shed light on many crucial aspects that must be considered for the rational development of prominent materials against pollutants.

## 2. Results and Discussion

### 2.1. Lattice Parameter Analysis

**Figure 2:**
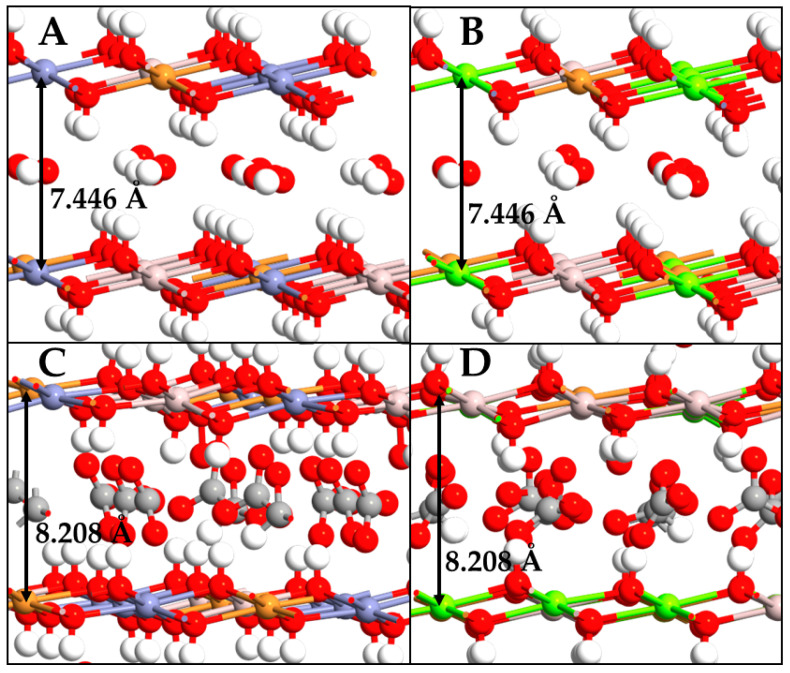
Front view of CuZnAl-OH^−^ (**A**), CuMgAl-OH^−^ (**B**), CuZnAl-CO_3_^2−^ (**C**), CuMgAl-CO_3_^2−^ (**D**). Atom types are reported following the color list of the previous figure.

The studied LDHs differ in the divalent cation acting as a structural element of the LDH system (Mg^2+^ or Zn^2+^). However, both exhibit a rhombohedral polymorph with space group R3m (166) [26], in which the metal hydroxide layers are separated by a specific spacing. After structural optimization, the anion type greatly influenced the interlayer spacing, which changed from 7.446 Å to 8.208 Å using OH^−^ and CO_3_^2−^ ions, respectively (Table 1). The hydroxyl ions were distributed in parallel in both the Mg- and Zn-LDH systems, while CO_3_^2−^ ions were tilted from each other, increasing the distance (Figure 2). Despite this obvious impact on spacing, the counterions did not have a significant influence on the octahedra layers, which instead showed only small arrangements in the space during the optimization process (Table 1). The only difference was due to the cation types involved; in fact, the Mg^2+^ ions induced a slight enlargement of the metal hydroxide layers along the x and y axes of the simulation box of the LDH unit cell, moving at 3.071 Å, whereas the Zn-LDH value was 3.062 Å. In further detail, for the CuZnAl-LDH system, the Cu-O, Al-O, and Zn-O bonds were 2.372 Å, 2.291 Å, and 2.353 Å, respectively; as regards the CuMgAl-LDH system, the Cu-O, Al-O, and Mg-O bonds were 2.341 Å, 2.224 Å, and 2.432 Å, respectively. The length of the Cu-O and Al-O bonds was slightly longer in CuZnAl-LDH, while the Mg-O showed a larger distance than Zn in their respective structures. It is important to note that, while the radii of the Zn^2+^ and Mg^2+^ ions are 0.88 Å and 0.87 Å, respectively, the strength of the chemical bond differs considering the two systems CuMgAl-LDH and CuZnAl-LDH.

The structural differences between the systems explain the discrepancies in the calculated formation energies. The number of hydrogen bonds (H bonds) generated by oxygen atoms of both OH^−^ and CO_3_^2−^ anions with hydrogen atoms belonging to the hydroxyl group of the LDHs was greater than that formed on one side in the same group as the metal hydroxide layers. This means that the system spontaneously pushes the oxygen atoms of the anions to be close enough to the hydroxyl hydrogen atoms of both sides’ basal layers to reach the distance for H bonds and thus exhibit a low formation energy. This parameter reflects the strength of the anion exchange capacity and the stability of the intercalation structure between the different layers.

The formation energies of systems with the same anions differed slightly, varying in several eV ranges. In this case, the important effect is attributed to the distance between the metal cations in the hydroxide layers. A more evident difference has been detected when comparing the formation energies of the systems with negative monovalent and divalent anions. In particular, the formation energy of CuZnAl has moved from −9.35 eV to −23.23 eV as a result of changing OH**^−^** with CO_3_^2^**^−^** ions, and a similar trend has been observed for CuMgAl, which exhibited a value of −6.92 eV and reached −21.46 eV as a result of moving from monovalent to divalent anions. This larger difference indicates that the binding energy is significantly influenced by the net charge of the interlayer anion since a higher number of electrons carried by the interlayer anion results in an increased formation energy. This means greater structural stability of LDHs, correlated with a stronger force between the interlayer anion and the basal LDH layer. Thus, anions with higher negative charge numbers are more likely to exchange for less-charged anions in the interlayer region of LDHs, and these results are in line with experimental data [27].

### 2.2. Intercalation Effects of NO_2_ and SO_2_

After the structural component of the four modeled systems was studied, their capacity to interstratify and capture NO_2_ and SO_2_ molecules was measured. For this purpose, these small molecules were manually inserted between the layers, and their orientations were monitored after the optimization process. As already reported, the systems were enriched with air pollutants to understand how stable they can be under these conditions and to assess the effect of intercalation from a qualitative and quantitative point of view. For these reasons, following intercalation, water molecules and counterions were excluded between layers of the systems. Since these molecules do not have equivalent molecular diameters along all axes, the spacing cannot be directly correlated [28], and we expect the same trend from DFT simulations.

**Figure 3:**
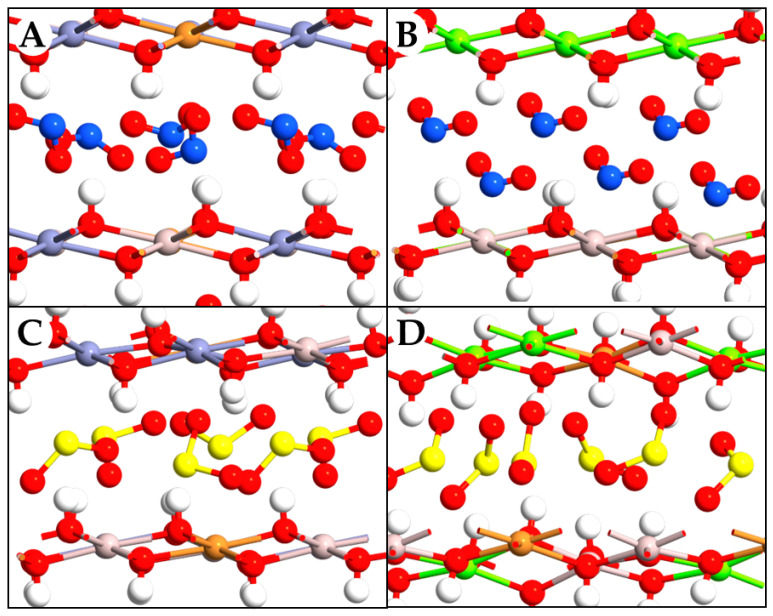
Front view of CuZnAl-NO_2_ (**A**), CuMgAl-NO_2_ (**B**), CuZnAl-SO_2_ (**C**), CuMgAl-SO_2_ (**D**). Atom types are reported following the color list of the previous figure, including N and S atoms in blue and yellow, respectively.

Focusing on NO_2_, when CuZnAl was used, NO_2_ molecules appeared efficiently placed between the layers. The reason is ascribed to the decreased distance between the cations along the layers, which also oriented the hydroxyl groups in opposite directions from each other, thus decreasing the repulsive phenomena with the NO_2_ oxygen atoms. Although NO_2_ is a natural molecule, its tridimensionality, together with its low dipole moment, led to an increased spacing, making intercalation in the CuZnAl LDH plausible. Furthermore, since NO_2_ could also be present as a dimer (hypoazotide), this would again increase the size of this molecule, canceling its dipole moment (Figure 3A).

A small difference has been observed with CuMgAl LDH, in which the O atoms of NO_2_ generated an electrostatic repulsion with the hydroxyl groups, deviating from a dense parallel arrangement between the layers and thus resulting in an increase in the interlayer distance. Similar results have been reported in the literature using NO_3_**^−^** as a counterion [29]. The greater ease of intercalation in LDH systems containing Zn^2+^ instead of Mg^2+^ is confirmed by the arrangement of the NO_2_ molecules between layers, which appears tilted compared to the arrangement adopted in the Mg-based system (Figure 3B). The molecules seem to be oriented to interact with both leaflets of the layers, and this orientation not only increases the stabilizing effect but prevents any dimerization of the NO_2_ molecules since the N atoms are oriented towards the LDH.

Concerning SO_2_, intercalation again increased the interlayer spacing, but to a lesser extent than for NO_2_. In the CuZnAl LDH, SO_2_ was highly intercalated between the layers, and all O atoms of SO_2_ formed H bonds with hydrogen atoms on the surface of the basal layers, both parallel and perpendicular (Figure 3C). Indeed, SO_2_ has a greater polarity compared to NO_2_, which is due to a higher electronegativity difference and a lower bond angle than NO_2_. This behavior allows SO_2_ to interact with the hydroxyl groups of the two layers much better than NO_2_, mimicking the behavior of the SO_4_^2^**^−^** ion [30], being able to be arranged either in a parallel or perpendicular direction in the available space. Furthermore, the CuZnAl system seems to intercalate the molecule better than the Mg system, since the greater distance of the latter components in the repeating unit caused a decrease in the H bonds between SO_2_ and LDH (Figure 3D). In summary, trapping NO_2_ seems to be more challenging, with significant results mainly with the CuZnAl LDH. Conversely, SO_2_ molecules could be intercalated with both LDH systems, exhibiting a more pronounced effect when the CuZnAl LDH is used.

### 2.3. Electronic Properties

**Figure 4:**
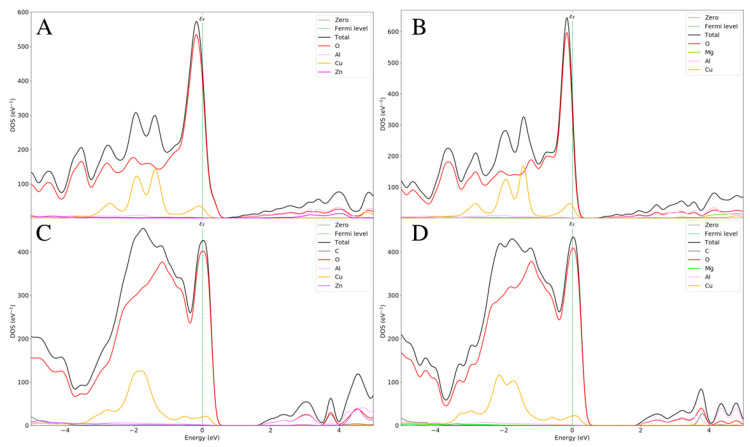
Partial and total density of states of CuZnAl-OH^−^ (**A**), CuMgAl-OH^−^ (**B**), CuZnAl-CO_3_^2−^ (**C**), and CuMgAl-CO_3_^2−^ (**D**).

The electronic properties of the different LDH systems with different counterions, with and without pollutants, have been calculated in terms of the density of states (DOS) and electron bandgap. The bulk properties of the semiconductor solid systems depend on these functions, and pollutants could induce advantageous variations in the electrical properties of LDHs. An analysis of the DOS of CuZnAl-OH**^−^**, CuMgAl-OH**^−^**, CuZnAl-CO_3_^2^**^−^**, and CuMgAl-CO_3_^2^**^−^** systems showed that the Valence Band Maximum (VBM) was obtained from the oxygen orbitals of the interlayer anions, and the oxygen orbitals of the hydroxyl groups belonged to the basal layer. This means that the most essential sites are the partial density of states of the valence band on the Fermi level, which is described by the interlayer anions rather than the hydroxyl group in the layer. This is also confirmed by the increase in VBM in the systems with CO_3_^2^**^−^** ions as the increasing charge of anions decreased the bandgap. On the other hand, the Conduction Band Minimum (CBM) of each pollutant-free system is composed mainly of the orbitals of Al^3+^ ions and partially, if present, of Zn^2+^ ions, which decrease the CBM. Considering these assumptions, the calculated bandgap values increase as follows: CuZnAl-OH^−^ (0.22 eV) < CuMgAl-OH**^−^** (0.73 eV) < CuZnAl-CO_3_^2^**^−^** (1.48 eV) < CuMgAl-CO_3_^2^**^−^** (1.69 eV) (Figure 4).

**Figure 5:**
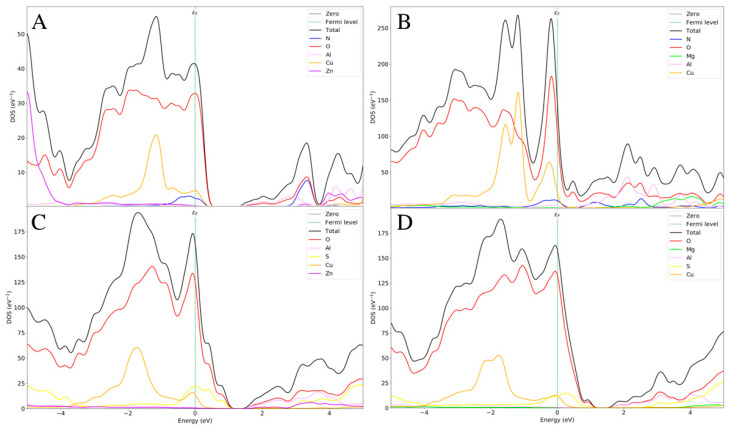
Partial and total density of states of Zn-Cu-Al/NO_2_ (**A**), Mg-Cu-Al/NO_2_ (**B**), Zn-Cu-Al/SO_2_ (**C**), and Mg-Cu-Al/SO_2_ (**D**).

The inclusion of the pollutants between the LDH layers led to essential changes in the electrical properties of the systems. In further detail, NO_2_ opens a wider bandgap in the ZnCuAl LDH, and the reason is mainly attributed to the NO_2_ molecules. The CBM is mainly constituted by N-2^p^ orbitals because the resonance energy involves the odd electron of the molecule; therefore, NO_2_ is reactive and then tends to dimerize. Furthermore, since NO_2_ has low ionization energy, it easily loses its odd electron and thus easily forms a hydronium cation NO_2_^+^. In addition, the N-2^s^ orbital contributes to VBM together with the Zn^2+^ orbitals, which exhibit an opposite trend compared to the CuZnAl with anions. In fact, Zn^2+^ highly participates in the description of much lower states, far from the Fermi level (Figure 5A). These two phenomena allow the maintenance of a wide bandgap, making the intercalation of NO_2_ in this system more than plausible.

When the Zn^2+^ is replaced with Mg^2+^, the electronic scenario looks completely different. Cu^2+^ seems to be much more involved in defining the states on the Fermi level. At the same time, the N and O orbitals completely close the bandgap providing a metallic behavior to this system when NO_2_ is included, resulting in the loss of the characteristic semiconducting properties (Figure 5B) [31,32,33,34,35,36].

When the SO_2_ intercalation was analyzed, a different trend from that in NO_2_ systems was observed. In this case, Zn^2+^ participates in the determination of the valence bands, while S and Cu^2+^ assist O in the determination of the VBM band, with an opened bandgap of 0.83 eV (Figure 5C). In the CuMgAl-SO_2_ system, the Mg^2+^ ions are mainly involved in the description of CBM; therefore, since the VBM is almost like the previous system, the change of the divalent cation has the only role of decreasing the CBM, with a final bandgap of 0.76 eV (Figure 5D). In summary, no evident changes in the electrical properties due to Zn^2+^ compared to Mg^2+^ are observed in the SO_2_ intercalation.

### 2.4. Charge Transfer

**Figure 6:**
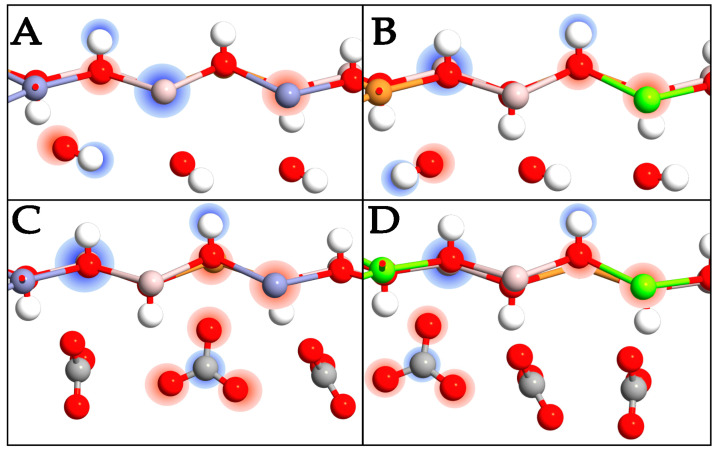
Front view of CuZnAl-OH^−^ (**A**), CuMgAl-OH^−^ (**B**), CuZnAl-CO_3_^2−^ (**C**), and CuMgAl-CO_3_^2−^ (**D**). Charge enrichment and charge depletion are shown in red and blue clouds, respectively. The charge density is highlighted only for one atom type and not for all to make the image clearer. The color scale for the atoms is the same as the previous figures.

**Figure 7:**
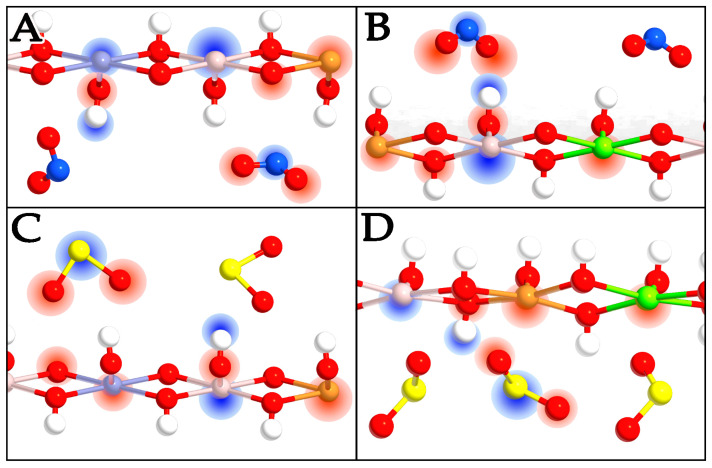
Front view of CuZnAl-NO_2_ (**A**), CuMgAl-NO_2_ (**B**), CuZnAl-SO_2_ (**C**), and CuMgAl-SO_2_ (**D**). Charge enrichment and charge depletion are shown in red and blue clouds, respectively. The charge density is highlighted only for one atom type and not for all to make the image clearer. The color scale for the atoms is the same as that in the previous figures.

Charge transfer has been calculated in all systems, focusing on the effects of pollutants. Each LDH system is made up of different components that attract themselves through electrostatic forces, meaning that one component has at least a partial negative charge and the other partner has a partial positive charge, acting as the electron acceptor and electron donor, respectively. The degree of charge transfer can be complete, and in this case, the complex can be classified as salt. In our cases, the charge transfer association is always weak, and the amplitude of interaction and local charge accumulation can be modulated by the type of molecule. In Figure 6 and Figure 7, the red regions indicate areas of charge accumulation, while the blue regions represent zones of charge depletion. From the results in the LDH layers with different anions, i.e., without pollutants, the charge transfer of metal cations is similar, meaning that the intercalated anions have only small effects on the electron distribution of the LDH metal hydroxide layers. The regions around Zn^2+^ ions are red, light red, and almost white, while the regions around Al^3+^ are blue; this means that the electronic density of Al^3+^ decreases evidently, and this effect can be attributed to the higher positive charge of Al^3+^ compared to Zn^2+^. Another interesting aspect is that the colored regions around Zn^2+^ and those of Al^3+^ are displayed separately without overlapping regions, indicating that the Zn-O and Al-O bonds have ionic characters (Figure 6A). In the models with Mg^2+^, the regions around these divalent ions have a slightly lighter shade of red than those with Zn^2+^ due to the lower electronegativity of Mg^2+^, while the regions around Al^3+^ remain at the same value of blue, and the colored regions around Mg^2+^ and those of Al^3+^ are again shown separately without overlapping (Figure 6B). In an analysis of the charge transfer phenomena of the layers and interlayers in all systems, the region around the H of OH^−^ groups is in blue, meaning that the electronic density of H decreases. On the other hand, the O atoms of the CO_3_^2−^ ions are surrounded by red regions, indicating an increase in electronic density (Figure 6C,D).

As expected, the intercalation of polluting compounds induces a charge redistribution, the amplitude of which depends on the intercalation efficiency of the molecules in each specific LDH type. In an analysis of the CuZnAl-NO_2_ system, there is an evident charge repartition on the NO_2_ molecule, favoring an accumulation on the O atoms to the detriment of the N species, which are depleted of charge (Figure 7A). Focusing on the layer, the charge on Zn^2+^ entities decreases while the charge of the Cu^2+^ ions increases. This variation allows the intercalation of NO_2_, which can form H bonds with the metal hydroxide layers, thus maintaining the peculiar characteristics of a semiconducting stable system.

In the CuMgAl-NO_2_ system, there is an increased charge on the Mg^2+^ ions, as well as a decrease on the Cu^2+^ entities. The general charge distribution and the alternation of electron-rich and electron-poor zones demonstrate global metallic behavior, which is the reason for the bandgap closure shown in the DOS analysis (Figure 7B).

In an analysis of the systems incorporating SO_2_, the Zn^2+^ ions do not show a charge depletion as in the case of NO_2_, while at the same time, the Cu^2+^ ions are not particularly charge enriched. Also, in this case, the electron density of the pollutant is almost completely localized on the oxygens, with an impoverishment on the S atom, which is even greater than that identified for N. This leads to a deeper charge distribution for SO_2_, which allows the electronic density of the molecule to avoid encountering repulsive factors with the LDH lattice while preserving the semiconducting properties (Figure 7C).

After the CuMgAl-SO_2_ LDH was analyzed, no peculiar effects on the density distribution were detected. The greater charge on Mg^2+^ weakens the electronic density of the OH^−^ polar groups, leading to less efficient interactions with SO_2_ (Figure 7D). It follows that, although the system maintains the necessary characteristics for intercalation, the presence of Mg^2+^ ions makes the interaction with SO_2_ more difficult than that of the Zn-Cu-Al LDH.

## 3. Materials and Methods

Quantum Atomistic Toolkit (Q-ATK) 2020.09-SP1 software [37] was used to model and simulate all the systems investigated. The Perdew–Burke–Ernzerhof (PBE) Generalized Gradient Approximation density functional [38] was used to quantify the electron exchange-correlation contribution, while the basis of the plane-wave (PW) method has been used to expand each single-particle wave function [39,40]. For each element, norm-conserving PseudoDojo pseudopotentials were used to approximate the core–shell [41].

All LDH systems were modeled and prepared from crystallographic data available in the literature, considering the assumption of the typical rhombohedral polymorphic structure of LDH, which is based on the *R3m* space group. Two different metal compositions were tested, considering CuZnAl, and CuMgAl LDH. In both systems, a metal ratio of 1:1:1 has been adopted, and each LDH system was analyzed together with two different counterions, OH^−^, and CO_3_^2−^ ions, leaving the systems free to move in space to accommodate atoms and then converge towards the optimal spacing between the layers. The counterions were then manually deleted and substituted with the same number of pollutant compounds to create plausible systems with NO_2_ and SO_2_ intercalated; finally, the optimization step was repeated.

Periodic boundary conditions were adopted along all axes to avoid problems with boundary effects caused by the finite size and at the same time to maintain high precision. Each model contains 4 LDH layers, with 3 explicit interlayer spaces and one located spatially halfway between the end and the beginning of the simulation box, to have perfect periodic reproducibility. Being rhombohedral systems, the layers do not have the same number of atoms, and this is due to the shape of the box that hosts a rhombohedral structure. The total number of octahedra is 32; this is also the number of LDH cations and, consequently, the number of OH^−^ counterions. Since the CO_3_^2−^ ion is divalent, 16 molecules were included between the layers. Finally, 4 molecules of NO_2_ and then of SO_2_ were considered. In consideration of a plausible intercalation, the counterions certainly could remain in space, even if to a lesser extent, as some water molecules do in any case; anyway, they have not been considered in the presence of NO_2_ and SO_2_ since the density of states would have been drastically affected by their presence otherwise. In this case, it would not have been possible to quantify the change in pollutant-induced electrical properties; however, half of the pollutant molecules that could be included were effectively inserted, and a negative potential gradient between the layers was also considered. From the optimization calculations, the energy cut-off was set to 1200 eV, with the Brillouin-zone integration settled at 15 × 15 × 15 k-points grid for each system [42], ensuring a total energy convergence of 5.0 × 10^−6^ eV/atom, with a maximum stress of 2.0 × 10^−2^ GPa, and the maximum displacement of 5.0 × 10^−4^ Å. The geometries of the chemical groups and molecules were not fixed, since the optimization was conducted with the cell symmetry preserved.

The DOS analysis was performed considering the energy range between −5 eV and 5 eV, with 0 eV corresponding to the Fermi level. The projections considered all the orbitals grouped for each element using the Gaussian spectrum method. The Monkhorst–Pack method was used as a grid type [43] with a periodic sampling of 22 × 22 × 22.

Charge transfer calculation was used to identify the electron donor–acceptor complexes, describing the supramolecular assembly of the complexes considering both molecules and ions. To highlight the areas with depletion or enrichment of charge more clearly, only one atom per species was reported with the cloud, and the same behavior was considered for the same types of atoms. The results were reported using the density plot type with a range between 0.026 and 0.46 Å^3^, corresponding to a depletion (represented by a blue cloud) and an enrichment (represented by a red cloud) of charge, respectively. Overall, the use of this level of theory is useful for faithfully reproducing short-range phenomena involving systems intercalated in lamellar layers, such as small molecules between the lamellae of LDHs [44,45].

## 4. Conclusions

The widespread use of different industrial processes and the fast-paced lifestyles that we are accustomed to have led to the massive production of small molecules which not only can be persistent in different environments but can pose significant risks to human health. In fact, prolonged exposure to small molecules such as NO_2_ and SO_2_ has been linked to a series of pathologies, which have become more prevalent in recent years. In this context, in this study, a computational method based on DFT has been adopted to model LDH-based systems as outstanding materials for co-intercalating NO_2_ and SO_2_ molecules, thus allowing their concentration in urban environments to be reduced or at least lowered. Our studies demonstrate how the CuZnAl system in a 1:1:1 molar ratio with CO_3_^2−^ as the counterion is extremely promising for the intercalation of NO_2_, while also preventing the possible dimerization of the molecule with itself. The structural studies, the density of states, and the charge transfer monitoring indicate that this system is extremely promising and selective for this purpose. As regards the intercalation of SO_2_, the simulations highlight how both CuZnAl and CuMgAl systems can be promising, with CuZnAl demonstrating better intercalation thanks to stronger interactions with the molecule, overcoming repulsive phenomena more efficiently.

This study also allowed the analyses of two-dimensional systems considering short- and long-range phenomena, which are difficult to observe from an experimental point of view. Considering the ease of preparation, the low manufacturing cost, and the tunability of the composition, the proposed approach opens the door to the investigation and the design of many possible LDH systems by appropriate modulation of their chemical–physical properties, making such systems extremely promising for the capture of pollutants in the next future.

## Figures and Tables

**Table 1:** Lattice parameters and the formation energy. The x, y, and z represent the axes of the space.

	CuZnAl-OH^−^	CuMgAl-OH^−^	CuZnAl-CO_3_^2−^	CuMgAl-CO_3_^2−^
**Cell parameters**	x 3.062 Å y 3.062 Å	x 3.071 Å y 3.071 Å	x 3.062 Å y 3.062 Å	x 3.071 Å y 3.071 Å
**Interlayer** **spacing**	z 7.446 Å	z 7.446 Å	z 8.208 Å	z 8.208 Å
**Formation** **energy**	−9.35 eV	−6.92 eV	−23.23 eV	−21.46 eV

## Data Availability

The original contributions presented in this study are included in the article; further inquiries can be directed to the corresponding author.
